# Tax4Fun: predicting functional profiles from metagenomic 16S rRNA data

**DOI:** 10.1093/bioinformatics/btv287

**Published:** 2015-05-07

**Authors:** Kathrin P. Aßhauer, Bernd Wemheuer, Rolf Daniel, Peter Meinicke

**Affiliations:** ^1^Department of Bioinformatics and; ^2^Department of Genomic and Applied Microbiology and Göttingen Genomics Laboratory, Institute of Microbiology and Genetics, Georg-August-University Göttingen, 37077 Göttingen, Germany

## Abstract

**Motivation:** The characterization of phylogenetic and functional diversity is a key element in the analysis of microbial communities. Amplicon-based sequencing of marker genes, such as 16S rRNA, is a powerful tool for assessing and comparing the structure of microbial communities at a high phylogenetic resolution. Because 16S rRNA sequencing is more cost-effective than whole metagenome shotgun sequencing, marker gene analysis is frequently used for broad studies that involve a large number of different samples. However, in comparison to shotgun sequencing approaches, insights into the functional capabilities of the community get lost when restricting the analysis to taxonomic assignment of 16S rRNA data.

**Results:** Tax4Fun is a software package that predicts the functional capabilities of microbial communities based on 16S rRNA datasets. We evaluated Tax4Fun on a range of paired metagenome/16S rRNA datasets to assess its performance. Our results indicate that Tax4Fun provides a good approximation to functional profiles obtained from metagenomic shotgun sequencing approaches.

**Availability and implementation:** Tax4Fun is an open-source R package and applicable to output as obtained from the SILVAngs web server or the application of QIIME with a SILVA database extension. Tax4Fun is freely available for download at http://tax4fun.gobics.de/.

**Contact:** kasshau@gwdg.de

**Supplementary information:** Supplementary data are available at *Bioinformatics* online.

## 1 Introduction

Amplicon-based sequencing of marker genes is widely used for large-scale studies that involve many different sampling sites or time series. The common 16S rRNA gene-based analysis is a powerful tool for assessing the phylogenetic distribution of a metagenome but does not provide insights into the communities metabolic potential. Therefore, the prediction of the functional capabilities of a microbial community based on marker gene data would be highly beneficial. As a particular difficulty of such a predictive approach for most organisms in marker gene databases the genome and therefore the functional repertoire is not known. For instance, the SILVA SSU rRNA database (Quast *et* *al.*, 2013) (SILVA 115 full release) contains 3 808 884 rRNA sequences whereas KEGG (Release 71.1) (Kanehisa *et* *al.*, 2014) only comprises 2982 complete prokaryotic genomes.

In a previous study (Aßhauer and Meinicke, 2013), we introduced a statistical method for predicting the metabolic profiles of a metagenome from its taxonomic composition using a linear combination of precomputed genomic reference profiles. A similar method has been proposed for inference of the community structure from remote sensing satellite image models (Larsen *et* *al.*, 2015). In this approach, the average EC number counts of all annotated genomes from a given taxonomic group, e.g. at order level, are used as reference for the linear combination of the community structure at this level. Recently, the PICRUSt approach was proposed to predict KEGG Ortholog (KO) functional profiles of microbial communities using 16S rRNA gene sequences (Langille *et* *al.*, 2013). PICRUSt infers unknown gene content by an extended ancestral state reconstruction algorithm. The algorithm uses a phylogenetic tree of 16S rRNA gene sequences to link operational taxonomic units (OTUs) with gene content. Thus, PICRUSt predictions depend on the topology of the tree and the distance to the next sequenced organism. Because a nearest neighbor within the tree topology always exists, PICRUSt links all OTUs, even if distances are large. This procedure can be problematic when analyzing microbial communities with a large proportion of so far not well-characterized phyla.

Here, we present Tax4Fun, a novel tool for functional community profiling based on 16S rRNA data. In Tax4Fun the linking of 16S rRNA gene sequences with the functional annotation of sequenced prokaryotic genomes is realized with a nearest neighbor identification based on a minimum 16S rRNA sequence similarity. Tax4Fun can be applied to the output of 16S rRNA analysis pipelines that can perform a mapping of 16S rRNA gene reads to SILVA. The results of Tax4Fun indicate that the correlation of functional predictions with the metagenome profile is higher as compared to the PICRUSt tool.

## 2 Implementation

Our method provides a prediction of functional profiles on the basis of SILVA-labeled OTU abundances. After preprocessing and clustering of the 16S rRNA sequencing reads the resulting OTUs have to be assigned to reference sequences in the SILVA database. The SILVA assignment counts are then transformed to functional profiles using Tax4Fun, which proceeds in three steps.

First, the SILVA-based 16S rRNA profile is transformed to a taxonomic profile of the prokaryotic KEGG organisms. The linear transformation is realized by a precomputed association matrix (see Supplementary Material section 2.1.1). Then, the estimated abundances of KEGG organisms are normalized by the 16S rRNA copy number obtained from the NCBI genome annotations. Finally, the normalized taxonomic abundances are used to linearly combine the precomputed functional profiles of the KEGG organisms for the prediction of the functional profile of the microbial community.

The organism-specific reference profiles are estimated with the same method as used for the Taxy-Pro reference profiles (Klingenberg *et* *al.*, 2013). For a fast computation of the organism-specific and metagenomic functional KEGG Ortholog (KO) profiles, we utilized UProC (Meinicke, 2015) and PAUDA (Huson and Xie, 2014), respectively (see Supplementary Material section 2.1.2).

## 3 Results

**Fig. 1.**
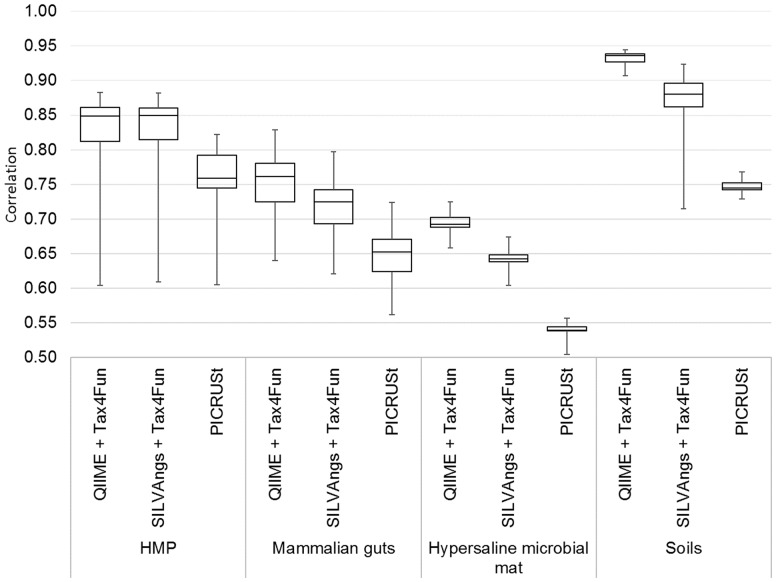
Spearman correlations between metagenomic and 16S-predicted functional profiles for comparison of Tax4Fun and PICRUSt on paired datasets from the human microbiome (HMP), mammalian guts, Guerrero Negro hypersaline microbial mat and soils

We applied Tax4Fun and PICRUSt to a collection of paired metagenome/16S rRNA datasets that have also been used in the original PICRUSt study (Fierer *et al.*, 2012; Harris *et al.*, 2013; Human Microbiome Project Consortium, 2012; Kunin *et* *al.*, 2008; Muegge *et* *al.*, 2011) (see Supplementary Material section 1.1). Before applying Tax4Fun, the SILVA-based 16S rRNA profiles were computed using the QIIME tool (Caporaso *et* *al.*, 2010) or the SILVAngs web server (Quast *et* *al.*, 2013), respectively (see Supplementary Material section 2.2 and 2.3). For each paired dataset, the Spearman correlation of the whole metagenome and the 16S rRNA-predicted relative KO abundance profile was calculated. For the computation of the correlation, we excluded for each dataset all KO profile dimensions that did not contribute any non-zero count in the functional profiles. The resulting correlation coefficients are shown in Figure 1 for the UProC-based functional profiles. Using Tax4Fun, the median of the correlation coefficient varies between 0.8706 (soils) and 0.6427 (Guerrero Negro hypersaline microbial mat).

In comparison with PICRUSt the correlation of Tax4Fun is significantly higher for all four datasets according to a nonparametric sign test (*P*-value < 0.001). Similar results are obtained using the PAUDA tool for estimation of the functional profiles (see Supplementary Material section 4.2).

Further, we compared the coverage of the analysis pipelines in terms of the fraction of reads that were classified by QIIME/SILVAngs and the percentage of OTUs that were mapped to KEGG organisms using Tax4Fun. Especially for the soil samples, we observed rather low fractions of 16S rRNA sequences that were finally used to predict the functional profiles (SILVAngs: 0.02%, Tax4Fun: 4.78%; QIIME: 95.21%, Tax4Fun: 55.36%). Contrary, the coverage for the human microbiome and mammalian guts datasets is rather high for both QIIME/SILVAngs and Tax4Fun (SILVAngs: 95%, Tax4Fun 95%).

In our study, the soil samples are probably the most complex communities under investigation. Further, our results revealed that members of the soil communities are poorly represented in the KEGG database. However, even when applying SILVAngs + Tax4Fun, the median of the correlation coefficients was rather high using merely a fraction of the reads to predict the functional profiles. Thus, a high correlation coefficient does not necessarily indicate the completeness of the estimated functional repertoire but rather provides a measure of correspondence between the whole metagenome and the 16S rRNA-predicted KO abundances.

Therefore, the prediction accuracy is not a function of sample diversity but rather depends on a good correspondence between organisms in genome and 16S rRNA databases. Even though a sample is very diverse good predictions can be obtained in case that many of the detected organisms or close relatives are available in both databases. In contrast, the predicted functional profile of samples with large fractions of unknown organisms can be expected to be incomplete due to the low coverage of database reference profiles. Thus, the coverage of the taxonomic assignments should always be inspected to check the reliability of the predictions, in particular when using SILVAngs. For all datasets, the coverage values are provided in the Supplementary Excel File.

## 4 Conclusion

Tax4Fun predicts the functional profile of a microbial community just from 16S rRNA sequence data. Our approach cannot replace whole metagenome profiling but is useful to supplement 16S rRNA analyses in metagenome pre-studies or in situations where shotgun sequencing is prohibitively expensive, e.g. for broad surveys in microbial ecology applications.

We evaluated our method on four paired data collections from different habitats and compared it to the PICRUSt tool. The results indicate a high correlation of the predicted Tax4Fun profiles with the corresponding functional profiles obtained from whole metagenome sequence data. Moreover, the results show that Tax4Fun outperforms PICRUSt on all test datasets. Additionally, our results revealed for all datasets a higher correlation between the metagenomic and 16S-predicted functional profiles when using UProC in comparison to the PAUDA tool (see Supplementary Material section 3 Figure S1–S3).

Although we provide functional reference profiles from both tools for Tax4Fun, we recommend the usage of Tax4Fun in combination with the UProC-based reference profiles for prediction because of the higher sensitivity of UProC.

Tax4Fun allows easy processing of the output from SILVAngs, QIIME or any other analysis pipeline using the SILVA database as reference. The implementation in R facilitates further statistical analyses of the Tax4Fun predictions, which can be processed within the same R environment.

## Funding

Grants from the Deutsche Forschungsgemeinschaft (ME 3138, to P.M. in part, TRR51, to R.D. in part).

*Conflict of Interest*: none declared.

## Supplementary Material

Supplementary Data
