# Supplementary material for: Tax4Fun: predicting functional profiles from metagenomic 16S rRNA data
Source: Bioinformatics. 2015 May 7;31(17):2882–4. doi: 10.1093/bioinformatics/btv287 (PMC4547618; doi:10.1093/bioinformatics/btv287)
Supplement: Supplementary Data [file supp_31_17_2882__index.html]

Tax4Fun: predicting functional profiles from metagenomic 16S rRNA data — Tax4Fun: predicting functional profiles from metagenomic 16S rRNA data — Supplementary Data 

# Tax4Fun: predicting functional profiles from metagenomic 16S rRNA data

## Supplementary Data

files

**Files in this Data Supplement:**

- Supplementary Material - pdf file
- Supplementary Excel File - xlsx file
