# Supplementary material for: Tax4Fun: predicting functional profiles from metagenomic 16S rRNA data
Source: Bioinformatics. 2015 May 7;31(17):2882–4. doi: 10.1093/bioinformatics/btv287 (PMC4547618; doi:10.1093/bioinformatics/btv287)
Supplement: Supplementary Data [file supp_btv287_Tax4Fun_SupplementalMaterial.pdf]

Supplementary Material for Aßhauer et al. "Tax4Fun: predicting functional profiles from metagenomic 16S rRNA data"

1 Supplementary Materials

1.1 Datasets

All publicly accessible amplicon and metagenome data sample files from the Human Microbiome Project (HMP) (Human Microbiome Project Consortium, 2012), mammalian guts (Muegge *et al.*, 2011), soils (Fierer *et al.*, 2012), Guerrero Negro hypersaline microbial mat (Kunin *et al.*, 2008; Harris *et al.*, 2013) were downloaded in December 2013. Due to the credit system of SILVAngs, we restricted the analysis of the HMP data to a subset of 49 samples for all taxonomic and functional profiling approaches. Subsequently the 16S profile from the amplicon data was estimated using SILVAngs (Quast *et al.*, 2013) and QIIME (Caporaso *et al.*, 2010) (see Supplementary Methods section 2.2 and 2.3). Finally, the functional profile of the microbial communities was predicted using the PICRUSt (Langille *et al.*, 2013) and Tax4Fun approach. In total, we were able to process 49 paired HMP samples, 56 mammalian guts samples, 13 paired soil samples, and 10 paired Guerrero Negro microbial mat samples (see Supplementary Excel File).

2 Supplementary Methods

2.1 Tax4Fun

2.1.1 Precomputation of the association matrix

In Tax4Fun, the linking of 16S rRNA gene sequences with the functional annotation of sequenced prokaryotic genomes is realized with a linear transformation of the SILVA-based 16S rRNA profile to a taxonomic profile based on the prokaryotic organisms in the KEGG database (Kanehisa and Goto, 2000; Kanehisa *et al.*, 2014). For the transformation, we precomputed an association matrix. The matrix is built from a BLASTN analysis where we extract 16S rRNA gene sequences of all prokaryotic organisms in KEGG (Release 64.0) and search them against the SILVA SSU Ref NR database (Release 115) (Quast *et al.*, 2013). For the assignment, we require a sufficient sequence similarity according to a threshold on the BLAST bitscore (>1500). A non-zero entry in this sparse matrix represents a valid assignment of a SILVA sequence identifier to one of the KEGG organisms. In case that K different KEGG organisms simultaneously

show significant hits for a SILVA 16S rRNA gene sequence the corresponding entries in the association matrix are set to $1/K$.

2.1.2 Precomputation of the functional reference profiles

Organism-specific functional profiles are precomputed for all prokaryotic genomes in KEGG. The genomes are downloaded and subsequently fragmented into overlapping reads simulating a two-fold coverage of the genomes as previously described in (Klingenberg *et al.*, 2013). To take different sequencing lengths into account, we generate overlapping reads of length 400 bp with 200 bp overlap for long read data and of length 100 bp with 50 bp overlap for short read data.

UProC (Meinicke, 2014) and PAUDA (Huson and Xie, 2014) are used for computation of the functional reference profiles in terms of KEGG Orthologs (KOs) of bacterial and archaeal origin. The UProC protein classification tool is executed in short read mode for the simulated short read data and in long read mode otherwise. The PAUDA homology search is performed in `--fast` mode with default parameters. In the case of multiple matches, only the best hit is considered.

In total, 6977 KOs of bacterial and archaeal origin are considered. By using UProC, we obtained 6644 (long reads) and 6671 (short reads) KO abundances for 1,943 genomes. By applying PAUDA, we captured only 3725 (long reads) and 3805 (short reads) KO abundances.

2.2 SILVAngs

First, 16S rRNA sequence data (see Supplemental Material section 1.1) was uploaded to SILVAngs. The SILVAngs analyses were performed using default parameters. Only the sequence type and sequencing technology were selected according to the specific characteristics of the amplicon data. Then, the results of the SILVAngs analysis were downloaded and the file:
`projectName---ssu---fingerprint----Total---sim_93---tax_silva---td_20.csv`
in the directory `results/ssu/tax_breakdown/fingerprint/` was used as input for the Tax4Fun approach.

2.3 QIIME (quantitative insights into microbial ecology)

Sequences were initially clustered into operational taxonomic units (OTUs) employing the `pick_otus.py` script implemented in the QIIME software package (v 1.8.0) (Caporaso *et al.*, 2010) with a similarity threshold of 0.97. Representative sequences (one per OTU) were selected with the `pick_rep_set.py` script. Taxonomy was assigned to these sequences using the `parallel_assign_taxonomy_blast.py` script and the most recent SILVA database (SSURef 115 NR) (Quast *et al.*, 2013) as reference. OTU tables were subsequently calculated using the `make_otu_table.py` script.

2.4 PICRUSt (phylogenetic investigation of communities by reconstruction of unobserved states)

Processed sequences were initially clustered into operational taxonomic units (OTUs) employing the `pick_closed_reference_otus.py` script implemented in the QIIME software

package with a similarity threshold of 0.97 and reverse strand matching enabled. The most recent greengenes reference database (gg_13_5) (DeSantis *et al.*, 2006) was used as reference for clustering. Obtained OTU tables were subsequently normalized by copy number using the `normalize_by_copy_number.py` script implemented in PICRUSt (v 1.0.0) (Langille *et al.*, 2013). Afterwards, functional predictions (KOs) and NSTI values were calculated based on normalized OTU tables employing the `predict_metagenomes.py` script. Here, the predicted functional trait abundances are inferred from 6,909 KO abundances of 2,590 sequenced bacterial and archaeal genomes from the IMG database (Markowitz *et al.*, 2012) v3.5.

3 Supplementary figures

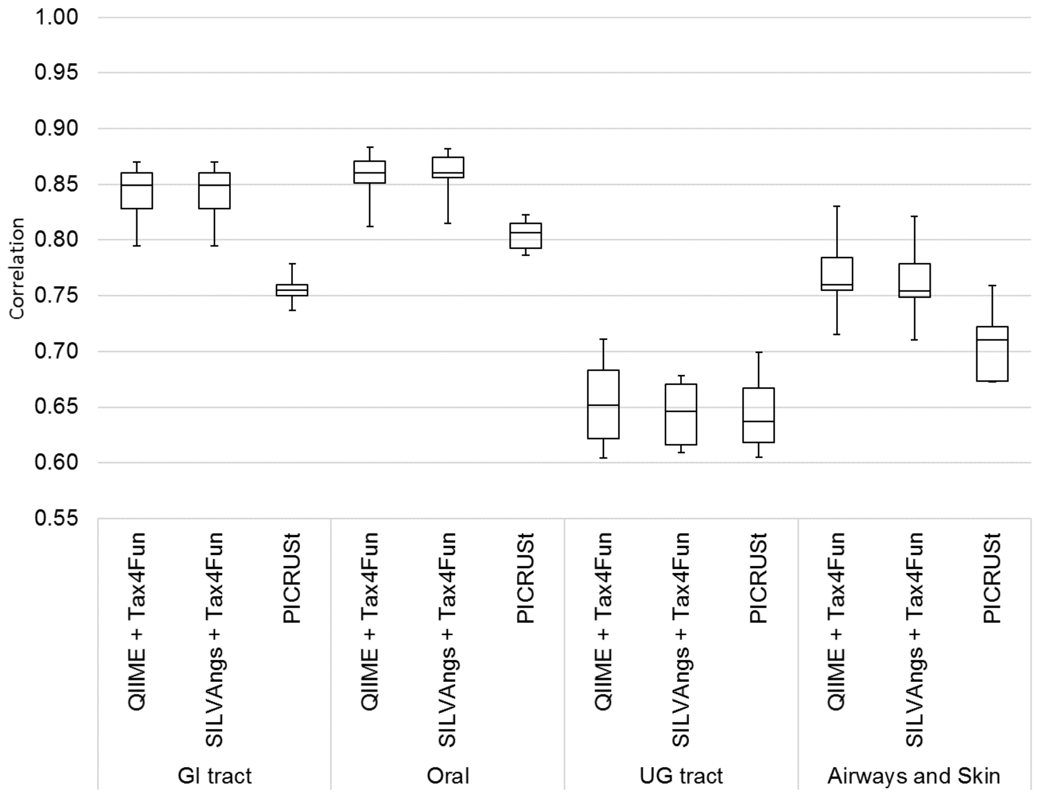

Figure 1: Spearman correlations between metagenomic and 16S-predicted functional profiles for comparison of Tax4Fun and PICRUSt on paired datasets from the human microbiome (HMP) across distinct body sites. Functional abundances of metagenomes and organism-specific reference profiles are based on UProC.

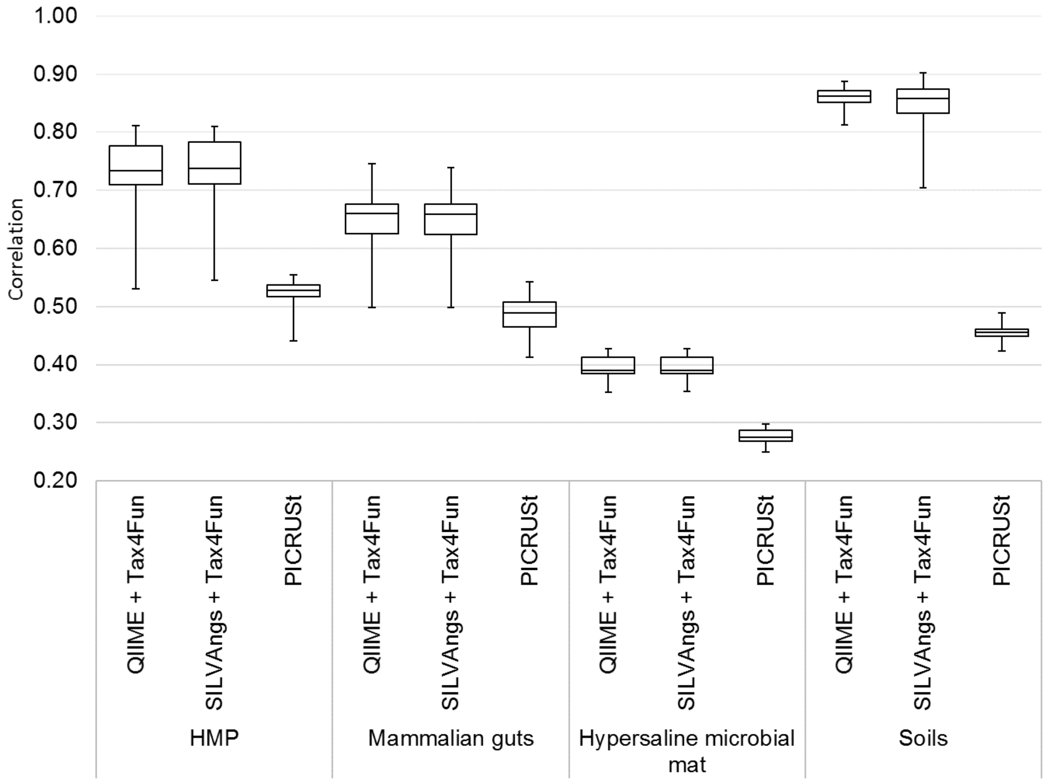

Figure 2: Spearman correlations between metagenomic and 16S-predicted functional profiles for comparison of Tax4Fun and PICRUSt on paired datasets from the human microbiome (HMP), mammalian guts, Guerrero Negro hypersaline microbial mat and soils. Functional abundances of metagenomes and organism-specific reference profiles are based on PAUDA.

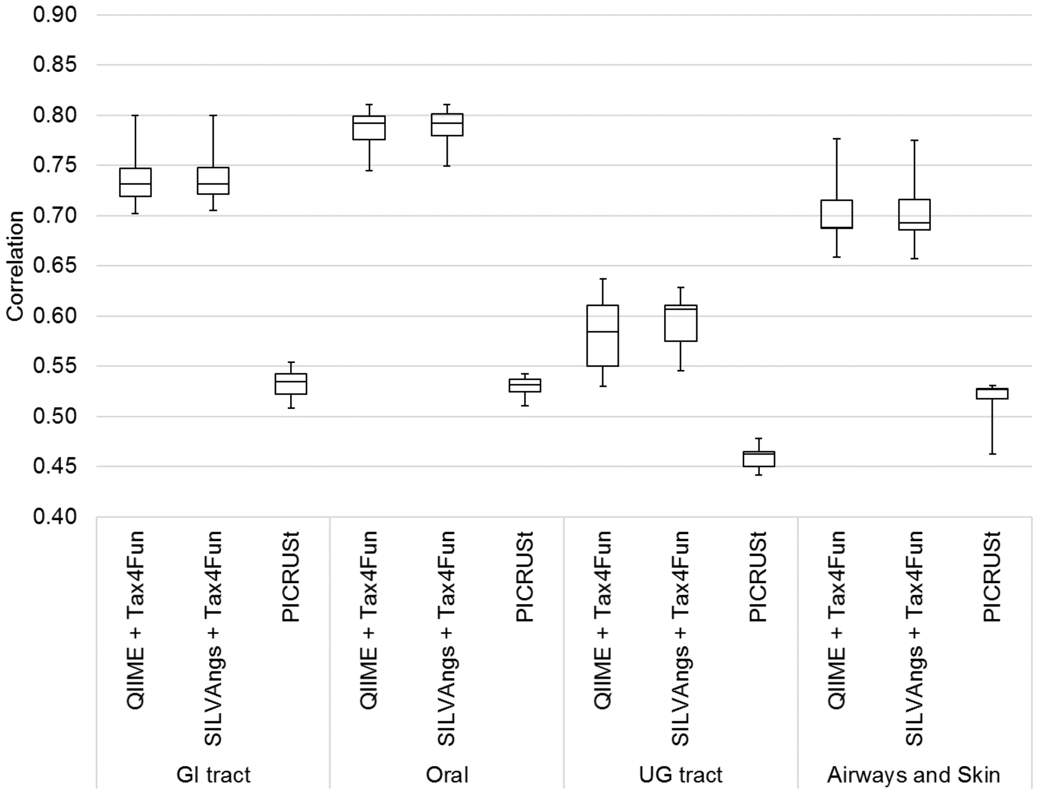

Figure 3: Spearman correlations between metagenomic and 16S-predicted functional profiles for comparison of Tax4Fun and PICRUSt on paired datasets from the human microbiome (HMP) across distinct body sites. Functional abundances of metagenomes and organism-specific reference profiles are based on PAUDA.

4 Supplementary Tables

4.1 Quality of the SILVA to KEGG assignment matrix

Because Tax4Fun was intended to realize a conservative mapping from SILVA 16S sequences to KEGG genomes, Tax4Fun requires a very high similarity over the whole 16S rRNA gene. For a sufficient sequence similarity, the BLASTN bitscore threshold was determined by inspecting the quality of the assignments at the phylum level. For different phyla, the percentage of correctly and incorrectly assigned SILVA entries to KEGG organisms was assessed. Table 1 shows the results for the selected BLASTN bitscore$>$1500.

By using a bitscore$>$1500 as threshold, a total of 292,978 SILVA identifiers could be assigned to 1943 out of 2045 KEGG organism. Reducing these 292,978 SILVA identifiers to unique taxonomic lineages, this resulted in 67,164 distinct taxonomic labels.

For example, in case of Acidobacteria, 98.28% of the SILVA identifiers are correctly assigned to KEGG organisms. Only 1.08% and 0.64% of the SILVA identifiers are incorrectly assigned to KEGG organisms of phylum Firmicutes or Proteobacteria. A similar high assignment quality is obtained for all major phyla.

Phylum in SILVA	Phylum in KEGG	Percentage of assignments
Acidobacteria (2916)	Acidobacteria	98.28%
	Firmicutes	1.08%
	Proteobacteria	0.64%
Actinobacteria (33887)	Actinobacteria	99.19%
	Firmicutes	0.01%
	Proteobacteria	0.80%
Aquificae (314)	Aquificae	99.75%
	Deinococcus-Thermus	0.25%
Armatimonadetes (1)	Proteobacteria	100.00%
Bacteroidetes (20188)	Bacteroidetes	98.07%
	Proteobacteria	1.79%
	Actinobacteria	0.10%
	Firmicutes	0.03%
	Verrucomicrobia	0.01%
Caldiserica (10)	Caldiserica	100.00%
Chlamydiae (161)	Chlamydiae	100.00%
Chlorobi (318)	Chlorobi	95.76%
	Ignavibacteriae	4.24%
Chloroflexi (326)	Chloroflexi	97.22%
	Proteobacteria	1.53%
	Other	0.96%
	Firmicutes	0.29%
Chrysiogenetes (10)	Chrysiogenetes	100.00%
Crenarchaeota (316)	Crenarchaeota	100.00%
Cyanobacteria (4598)	Cyanobacteria	99.95%
	Acidobacteria	0.05%
Deferribacteres (36)	Deferribacteres	78.65%
	Proteobacteria	21.35%
Deinococcus-Thermus (555)	Deinococcus-Thermus	100.00%
Dictyoglomi (9)	Dictyoglomi	100.00%
Elusimicrobia (98)	Elusimicrobia	100.00%
Euryarchaeota (5252)	Euryarchaeota	99.18%
	Firmicutes	0.57%
	Other	0.24%
	Thaumarchaeota	0.01%

Fibrobacteres (70)	Fibrobacteres	99.00%
	Proteobacteria	1.00%
Firmicutes (83887)	Firmicutes	99.90%
	Proteobacteria	0.09%
	Actinobacteria	0.01%
Fusobacteria (1819)	Fusobacteria	95.94%
	Proteobacteria	2.32%
	Firmicutes	1.74%
	Ignavibacteriae	0.01%
Gemmatimonadetes (224)	Gemmatimonadetes	100.00%
Korarchaeota (26)	Korarchaeota	100.00%
Nanoarchaeota (1)	Proteobacteria	100.00%
Nitrospirae (444)	Nitrospirae	92.27%
	Other	7.38%
	Firmicutes	0.35%
Planctomycetes (422)	Planctomycetes	100.00%
Proteobacteria (132058)	Proteobacteria	99.98%
	Firmicutes	0.01%
	Actinobacteria	0.01%
Spirochaetae (1353)	Spirochaetes	99.98%
	Proteobacteria	0.02%
Synergistetes (158)	Synergistetes	100.00%
Tenericutes (786)	Tenericutes	100.00%
Thaumarchaeota (1840)	Thaumarchaeota	99.97%
	Crenarchaeota	0.03%
Thermodesulfobacteria (44)	Thermodesulfobacteria	100.00%
Thermotogae (188)	Thermotogae	100.00%
Verrucomicrobia (660)	Verrucomicrobia	99.61%
	Proteobacteria	0.39%

Table 1: Assessment of the assignment quality of SILVA entries to KEGG organisms using a bitscore >1500 in the BLASTN analysis. The table shows for different phyla the percentage of SILVA 16S rRNA entries which are assigned to KEGG organisms collated at the phylum level. Values represent rounded percentages and entries lower than 0.01% are omitted. Number in brackets indicate the number of SILVA identifiers for the respective phylum.

4.2 Statistical testing

	UProC		PAUDA	
Dataset	QIIME + Tax4Fun vs. PICRUSt	SILVAngs + Tax4Fun vs. PICRUSt	QIIME + Tax4Fun vs. PICRUSt	SILVAngs + Tax4Fun vs. PICRUSt
HMP	1.78E-013	8.23E-010	3.55E-015	3.55E-015
Mammalian gut	2.78E-017	2.78E-017	2.78E-017	2.78E-017
Guerrero Negro	1.95E-003	1.95E-003	1.95E-003	1.95E-003
Soil	1.83E-003	1.22E-004	1.22E-004	1.22E-004

Table 2: P-values from nonparametric statistical testing for significant differences between correlation coefficients of different methods. Results are shown for metagenome functional abundances and organism-specific reference profiles as estimated by UProC and PAUDA for all four datasets.

4.3 Quality survey of the prediction methods

For all taxonomic and functional annotation approaches, the quality of the predictions was assessed. For the functional metagenome annotation using UProC and PAUDA the Fraction of Sequences Unexplained (FSU) (Klingenberg *et al.*, 2013) was calculated. The coverage of the QIIME and the SILVAngs analysis pipelines was assessed in terms of the fraction of reads that could be classified by QIIME/SILVAngs. For Tax4Fun, we calculated the Fraction of Taxonomic units Unexplained (FTU). The FTU measures the fraction of sequences assigned to taxonomic units that can not be mapped to KEGG organisms using the association matrix. For PICRUSt, we used the weighted nearest sequenced taxon index (NSTI) for the quality survey. For all datasets and profiling methods, the quality values can be obtained from the Supplementary Excel File ”SupplementalExcelFile_QualitySurvey.xlsx”.

References

Caporaso, J. G., *et al.* (2010). QIIME allows analysis of high-throughput community sequencing data. *Nat Methods*, **7**(5), 335–336.

DeSantis, T. Z., *et al.* (2006). Greengenes, a Chimera-Checked 16S rRNA Gene Database and Workbench Compatible with ARB. *Applied and Environmental Microbiology*, **72**(7), 5069–5072.

Fierer, N., *et al.* (2012). Cross-biome metagenomic analyses of soil microbial communities and their functional attributes. *Proc Natl Acad Sci U S A*, **109**(52), 21390–21395.

Harris, J. K., *et al.* (2013). Phylogenetic stratigraphy in the Guerrero Negro hypersaline microbial mat. *ISME J*, **7**(1), 50–60.

Human Microbiome Project Consortium (2012). Structure, function and diversity of the healthy human microbiome. *Nature*, **486**(7402), 207–214.

Huson, D. H. and Xie, C. (2014). A poor man's BLASTX–high-throughput metagenomic protein database search using PAUDA. *Bioinformatics*, **30**(1), 38–39.

Kanehisa, M. and Goto, S. (2000). KEGG: kyoto encyclopedia of genes and genomes. *Nucleic Acids Res*, **28**(1), 27–30.

Kanehisa, M., *et al.* (2014). Data, information, knowledge and principle: back to metabolism in KEGG. *Nucleic Acids Res*, **42**(Database issue), D199–D205.

Klingenberg, H., *et al.* (2013). Protein signature-based estimation of metagenomic abundances including all domains of life and viruses. *Bioinformatics*, **29**(8), 973–980.

Kunin, V., *et al.* (2008). Millimeter-scale genetic gradients and community-level molecular convergence in a hypersaline microbial mat. *Mol Syst Biol*, **4**, 198.

Langille, M. G. I., *et al.* (2013). Predictive functional profiling of microbial communities using 16S rRNA marker gene sequences. *Nat Biotechnol*, **31**(9), 814–821.

Markowitz, V. M., *et al.* (2012). Img: the integrated microbial genomes database and comparative analysis system. *Nucleic Acids Research*, **40**(D1), D115–D122.

Meinicke, P. (2014). UProC: tools for ultra-fast protein domain classification. *Bioinformatics*.

Muegge, B. D., *et al.* (2011). Diet drives convergence in gut microbiome functions across mammalian phylogeny and within humans. *Science*, **332**(6032), 970–974.

Quast, C., *et al.* (2013). The SILVA ribosomal RNA gene database project: improved data processing and web-based tools. *Nucleic Acids Research*, **41**(D1), D590–D596.
